# A Strange Case of Dissociative Identity Disorder: Are There Any Triggers?

**DOI:** 10.7759/cureus.2957

**Published:** 2018-07-10

**Authors:** Muhammad Awais Rehan, Annapurna Kuppa, Abhilasha Ahuja, Shazra Khalid, Nishita Patel, Firman Sandiyah Budi Cardi, Viraj V Joshi, Amna Khalid, Hassaan Tohid

**Affiliations:** 1 Department of Pathology, Khawaja Muhammad Safdar Medical College, Sialkot, PAK; 2 Department of Research, University of Bonn/the Johns Hopkins University, Baltimore, USA; 3 Department of Research, California Institute of Behavioral Neurosciences & Psychology, Fairfield, USA; 4 Department of Research, California Institute of Behavioral Neurosciences & Psychology, Karachi, PAK; 5 Research, California Institute of Behavioral Neurosciences & Psychology, Fairfield, USA; 6 Internal Medicine, Icahn School of Medicine, Mount Sinai/Queens Hospital Center, New York, USA; 7 Neurology, Center for Mind & Brain, University of California, Davis, Fairfield, USA

**Keywords:** dissociation, did, personality disorders

## Abstract

We discuss a strange case of dissociative identity disorder, also known as multiple personality disorder. This article describes the case of a 55-year-old Caucasian woman with a history of substance use disorder with seven personalities. The patient describes a couple of triggers for her condition. More research is needed to understand these triggers.

## Introduction

Dissociative identity disorder (DID), or dissociative personality disorder, is the presence of at least two varied personalities in one person [1-2]. Thus, it is also referred to as multiple personality disorder [3]. There are several conditions found to be associated with this disorder, including depression, self-harm, post-traumatic stress disorder (PTSD), substance use disorder, borderline personality disorder or anxiety [4-5], and conversion or somatoform disorder [6]. DID also includes the unexplained loss of personal information from one's memory [1]. The estimated DID prevalence around the globe is about 5% among the inpatient psychiatric population, 2%–3% among outpatients, and 1% in the general population [7-8].

In this case study, we present an interesting case of DID with triggers. The association of triggers with DID is not well-studied and understood. We hope that this case study will help unearth the possible association of DID with triggers like stress and substance use disorder.

## Case presentation

Here, we present a case of a 55-year-old Caucasian female with a history of substance use disorder and a comorbid bipolar disorder, who presented to the local general hospital with a history of the fragmentation of a single personality into different personalities under emotional stress and under the influence of a drug. Multiple aspects of her personalities were reported, including the following:  a personality of a seven-year-old child, a personality that would behave as a teenager, and another that acted like a male person in addition to her normal 55-year-old personality. She reported that she had been constantly dominated by her alternate personalities and became aware of their existence when people around her informed her, usually after a situation ended. She reported that stressful situations and substance abuse could aggravate the fragmentation of her personality. This was found to be mostly an involuntary phenomenon with seldom memory of the event.

While transitioning between these personalities, she was found to be violent even to people who were close to her. This could range from being suicidal to homicidal for which she was arrested twice in the past. She had to be isolated and restrained by being locked in a room and calling the police. As a result, she was hospitalized in a mental institution for a significant period at least two to three times in the past. Under the influence of stress or substances like marijuana or cocaine, her personality would split into various personalities. These states were very different from one another in terms of age or gender.

One of her alternate personalities behaved as a seven-year-old child and would show the same interests and choices that included becoming moody or a self-arrogant personality. While in these states, she could hurt herself or had weeping spells if her wants were not met.

Another personality acted as a teenager with some sharp choices and dressing. Increase in substance abuse, alcohol use, and smoking would lead to multiple cases of fights or homicidal attacks, with some incidents of self-harming events. Multiple scars were found on the dorsal side of her right hand. Her speech was found to be pressured and she would repeat the same words/ conversations.

The next personality was diagnosed to be a temporary transition to the opposite gender (a male). There was a change in voice and behavior. This included male dressing, language, a perception of male body parts, choices of friends, and attraction towards females, including sexual behavior.

The normal state of a 55-year aged female was the default personality that made her feel most comfortable. She reported that she had anxiety during a personality state transition, as it could occur at any time, and involuntarily, but mostly in stressful situations and during substance abuse. More violent and harmful events were reported when someone tried to meet the patient alone rather than in a group.

The treatment included psychotherapy with cognitive behavioral therapy addressing stress and substance use disorder. The psychotherapeutic treatment lasted for at least six months. The dual treatment of drug therapy was also involved to calm her down. The patient was prescribed escitalopram to reduce her anxiety symptoms. She believed that the anxiety pills were really helpful. After six months, the patient's condition was not drastically different. However, she believed her stress was getting better. The patient was further followed up for the next one year and the treatment continues to date. 

## Discussion

Dissociative identity disorder (DID) is a severe condition characterized by a marked discontinuity in the identity of an individual, with fragmentation into two or more distinct personality states, which alternately take control of the individual. It reflects a loss of the ability to assimilate various aspects of one’s identity, memory, as well as consciousness into a single multidimensional self.

The primary identity is the individual’s original identity that carries their given name and is passive, dependent, and usually guilty and depressed. The alternate personality states, when in control, have a distinct history and identity, with their own pattern of self-perception. These states are referred to as “Alters” and have different characteristics compared with the primary identity. These characteristics could include the name, age, gender, functions, mood, memories, and vocabulary, along with other traits. The emergence of a particular episode generally occurs due to a certain stressor.

Symptoms

According to the Diagnostic and Statistical Manual of Mental Disorders, 5th edition (DSM 5) published in 2013, the symptoms of dissociative identity disorder include:

●       The individual experiences two or more distinct identities or personality states, each with its own pattern of perceiving, relating to, and thinking about the self and the world.

●       The disruption in identity involves a change in sense of self, loss of personal agency, and alterations in effect, behavior, consciousness, memory, perception, cognition, and/or sensory-motor function.

●       Frequent gaps in memories of personal history, including the distant and recent past as well as everyday events. These gaps are not consistent with the expected normal forgetfulness.

●       Significant distress and impairment in the level of functioning because of the symptoms.

●       The disturbance cannot be attributed to the physiological effects of a substance or another medical condition.

More than 70% of people with DID have attempted suicide, and self-injurious behavior is common among this population [2]. Treatment is crucial to improving quality of life and preventing suicide attempts. Cognitive Behavioral Therapy (CBT) could be used as a mode of treatment for these patients [9].

Causes

The cause of DID is not completely understood. It is believed to be due to severe physical and sexual abuse, particularly during childhood. Among those with DID in the U.S., Canada, and Europe, approximately 90% report a history of abuse in childhood.

Our findings

The case study discussed here describes a strange case of DID where the patient had triggers of the DID episodes. Every time the patient had an episode of a new personality, she had triggers like a migraine patient would have before an episode. She was either under the influence of a drug or she had emotional stress, which led to a personality change. The strength of this case study is that we have an idea that probably stress and drug use can lead to DID symptoms. However, we don't yet know if this change in personality is only due to marijuana and cocaine or can be due to any drug use. We also need more understanding of whether her episodes were triggered when she had either one of these triggers or is it necessary to have both triggers at the same time to develop DID symptoms.

## Conclusions

In the described case, the patient had dissociative identity disorder. Her condition was reported to be associated with any emotional stress and substance use disorder. The transition to a different personality was involuntarily and, most of the time, the patient was not aware of the transition or had any memory of the event. Most patients with this condition are informed by nearby observers about the personality state shift. Post-event confusion and guilt is also reported sometimes. Avoidance of triggers like stress and substance abuse may help in unpredicted personality fragmentations into multiple personality states. However, more research is needed to understand these possible triggers better.
